# P076 Spontaneous remission of polymyalgia rheumatica without treatment

**DOI:** 10.1093/rap/rkag113.106

**Published:** 2026-11-03

**Authors:** Tony Yap, Mariam Malik

**Affiliations:** Basingstoke and North Hampshire Hospital, Basingstoke, United Kingdom; Basingstoke and North Hampshire Hospital, Basingstoke, United Kingdom

## Abstract

**Introduction:**

Polymyalgia rheumatica (PMR) is a common inflammatory disease with rapid onset inflammatory-type pain and stiffness around proximal girdles often with systemic symptoms, commonly above 50-years-old. There is usually an increase in erythrocyte sedimentation rate (ESR) and/or C-reactive protein (CRP). An ESR above 40mm/hr can be diagnostic and prognosticating.

About 15% of PMR patients have concurrent giant cell arteritis (GCA) which can have sight-threatening and ischaemic complications urging early diagnosis and treatment. Management of both include high-dose glucocorticoids with subsequent tapering regime.

We present a case of PMR that was managed conservatively, achieving spontaneous resolution of symptoms and biochemical picture.

**Case description:**

We present the case of a 70-year-old lady with background of hypothyroidism and hypertension. She reported fever and cough a week after a dental filling about 2 months prior to presentation to rheumatology. Initial bloods at that time showed a raised CRP at 196mg/L and ESR of 106mm/hr. Her white cell count was raised at 12.9x109/L, Hb was 109 g/L and thrombocytosis at 675x109/L.

By the time she was referred to Rheumatology, her initial symptoms had mostly resolved with residual myalgia at about 70% of baseline, fatigue and weight loss of 3-4 kg over a few weeks which had stabilised. She reported about an hour of early morning stiffness.

She denied any visual symptoms, headaches or infective symptoms. She was recently seen by opticians due to floaters and cataracts were detected. There was no clinical synovitis or any signs of GCA. She had good range of movement of both shoulders and no proximal weakness. Her temporal and axillary ultrasound did not show any evidence of vasculitis. Blood tests were repeated and these showed a CRP of 67mg/L from 196mg/L and ESR 73mm/hr from 106mm/hr. RF, anti-CCP, ANA and ANCA were negative. Myeloma screen and thyroid function normal. Hepatitis B and C, HIV, CMV, EBV, Parvovirus and blood cultures were negative. Echocardiogram was normal.

PET CT showed mild increased tracer uptake over interspinous bursae and adjacent to the greater trochanters and ischial tuberosities; findings were consistent with PMR.

Steroids were offered but she was not keen and preferred taking herbal medications - Boswellia, ginger and curcumin. The risks of foregoing treatment were discussed, including the potential development of giant cell arteritis. Safety-net advice was provided and follow up arranged for careful monitoring.

At about 9 months review since onset, she reported resolution of her symptoms with normalisation of the biochemical profile.

**Discussion:**

Polymyalgia rheumatica can occasionally go into remission without treatment, but this is thought to be uncommon. The condition is usually treated as symptoms can be severe and there is a risk of GCA. Untreated PMR can lead to prolonged pain, stiffness, fatigue, loss of function, and reduced quality of life. Glucocorticoids remain the first-line treatment and typically provide rapid symptom relief. However, the potential adverse effects of prolonged corticosteroid therapy, which commonly requires tapering over approximately 12 months, may significantly influence patient preference. Shared decision-making is therefore essential, with counselling addressing both the benefits of glucocorticoid therapy and the risks associated with declining treatment, including the development or unmasking of GCA and its potentially serious complications. Simultaneously, alternative diagnoses, including vasculitis and malignancy, should be excluded. In this case, whole-body PET-CT was undertaken to minimise diagnostic uncertainty and mimics.

Although PMR is generally regarded as a self-limiting condition, evidence regarding its natural history without treatment remains limited, with symptom duration reported to range from 1 to 5 years. While spontaneous remission is possible, it is not predictable, and current practice is to treat PMR rather than wait for it to resolve on its own.

Evidence for non-pharmacological or alternative therapies in PMR is similarly limited. One small comparative study demonstrated comparable improvements in pain and stiffness between pharmacological and non-pharmacological management, although disease flares occurred more frequently in patients who did not receive glucocorticoids.

**Key learning points:**

1. Polymyalgia rheumatica (PMR) is an inflammatory condition that typically affects adults over the age of 50. It is characterised by pain and stiffness in the shoulders and pelvic girdle and is typically associated with an acute phase response. The mainstay of treatment is low-dose glucocorticoids (such as prednisolone), which often produce marked improvement within a few days.

2. Investigative pathways for GCA, malignancy, connective tissue disease and myopathy with thorough examination must be undertaken to ensure no underlying diagnoses mimicking PMR are missed which may lead to potential morbidity and mortality.

3. PMR can occasionally go into remission without treatment, but this is thought to be uncommon, and the condition is usually treated because symptoms can be severe and there is an associated risk of giant cell arteritis. Historically, untreated cases have been reported to remit spontaneously, but high-quality data on the untreated natural history are limited because most patients receive treatment soon after diagnosis.

4. This case underscores the importance of open doctor-patient communication and partnership, presenting information such as rationale, risks, benefits of treatment and consequences of not treating for an informed decision to be reached. Concerns about side effects should also be addressed and how they can be mitigated to improve tolerance and compliance, such as the use of proton pump inhibitors and bone protection.

5. Complementary herbal remedies may have a beneficial role, although the evidence remains limited. More robust research studies are needed to evaluate their efficacy and safety.

6. Regardless of the chosen management, providing safety-net advice to the patient and arranging regular follow-ups are important to monitor for complications and evolving symptoms that may necessitate further investigations or revisiting management options.

